# A case/non-case study of a national pharmacovigilance database to explore drug-induced acute kidney injury

**DOI:** 10.1007/s11096-025-01940-0

**Published:** 2025-05-26

**Authors:** Catarina Luz Oliveira, Fernando Fernandez-Llimos, Filipa Alves da Costa, João Pedro Aguiar, Filipa Duarte-Ramos

**Affiliations:** 1 iMED, Research Institute for Medicines, Faculty of Pharmacy, Universidade de Lisboa, Av. Professor Gama Pinto, 1649-003 Lisbon, Portugal; 2 Hospital Vila-Franca de Xira, Vila Franca de Xira, Portugal; 3 UCIBIO–Applied Molecular Biosciences Unit, i4HB–Institute for Health and Bioeconomy, Laboratory of Pharmacology, Faculty of Pharmacy, Universidade of Porto, Porto, Portugal; 4 EPIUnit, Epidemiology Unit, Laboratory for Integrative and Translational Research in Population Health (ITR), Universidade do Porto, Porto, Portugal

**Keywords:** Acute kidney injury, Adverse drug reaction reporting systems, Patient safety, Pharmacoepidemiology, Pharmacovigilance, Risk management

## Abstract

**Background:**

Monitoring safety throughout a medicine’s lifecycle is essential. Pharmacovigilance systems are rich sources contributing to this aim in a real world context.

**Aim:**

To identify and estimate disproportionality rates associated with the drugs that are most frequently reported to induce acute kidney injury (AKI).

**Method:**

A case/non-case study was conducted, using data extracted in 2022 from the Portuguese National Pharmacovigilance Database for the period between 01/01/2009 and 12/31/2020. Cases were identified using the ‘Acute Renal Failure’ standardized MedDRA query, all remaining reports were considered non-cases, and a random sample without replacement of 4 non-cases per case was extracted. Data were expressed as the reporting odds ratio (ROR) and the 95% confidence interval.

**Results:**

During this 11-year period, 352 AKI cases were identified, representing 0.7% of the 53,505 reports received. A total of 559 different drugs were considered 'suspect' in these AKI cases. Three therapeutic subgroups (ATC2) showed a significant ROR: antithrombotic agents (ROR 6.72; 95% CI 2.23–20.22), antivirals for systemic use (ROR 4.02; 95% CI 2.76–5.87), and antineoplastic drugs (ROR 2.14; 95% CI 1.48–3.11). Additionally, we identified individual drugs with significant RORs where no class effect was observed, namely mycophenolic acid, ciclosporin, tacrolimus, simvastatin, prednisolone, vancomycin, and deferasirox. In total, eleven drugs were identified as potentially associated with the occurrence of AKI.

**Conclusion:**

This study highlights the importance of clinical pharmacy activities in closely monitoring renal function of people with known risk factors or those prescribed medications known to increase the risk of AKI. Some of the medications identified require further investigation to validate their association with AKI.

**Supplementary Information:**

The online version contains supplementary material available at 10.1007/s11096-025-01940-0.

## Impact statements

- Reports made to the Portuguese national pharmacovigilance database suggest antithrombotic agents, antivirals for systemic use, and antineoplastic drugs to be associated with the occurrence of acute kidney injury.
- This study highlights the importance of clinical pharmacy routine monitoring of medicines with higher reporting odds, including dabigatran etexilate, simvastatin, emtricitabine, and mycophenolic acid
- An important proportion of acute kidney injuries can potentially be prevented by clinical pharmacy interventions, namely through medicines optimization.

## Introduction

Acute kidney injury (AKI) is a serious complication often seen in hospitalized patients, commonly triggered by nephrotoxic drugs [1, 2]. While the development of AKI in the community is estimated to happen in approximately 20 to 200 cases per million population, in hospitalized patients, this estimate increases to 10–15% and in patients admitted to the intensive care unit, it is described as exceeding 50% [3]. This increase in risk of AKI observed during the hospital stay justifies enhanced investments in clinical pharmacy interventions that may prevent its occurrence but also its progression to chronic renal failure, which is a well-recognized risk [2].

Different definitions and respective criteria for AKI have been used, such as those developed by the “Acute Kidney Injury Network”, known as AKIN, the “Kidney Disease: Improving Global Results”, known as KDIGO and the “Risk, Injury, Failure, Loss, End-stage kidney disease”, known as RIFLE. KDIGO clinical practice guidelines are the most widely used and apply to children [4]. In these guidelines, AKI is defined as an increase in serum creatinine (SCr) by ≥ 0.3 mg/dL (≥ 26.5 µmol/l) within 48 h; or an increase in SCr to ≥ 1.5 times baseline, which is known or presumed to have occurred within the preceding 7 days; or a urine volume < 0.5 ml/kg/h for 6 h [5].

Drug-related kidney injury is estimated to be responsible for 19–26% of all AKI cases in hospitalised patients [3], and for a significantly higher proportion among critically ill patients admitted to intensive care units [6].

Various studies have reported different drugs that induce AKI, though many focus on single drug classes, showing variability across countries [7–10]. A French national pharmacovigilance database (FPVD) study found AKI in 3.2% of drug-related reports. The most frequently implicated classes were antibacterial agents (29.5%), diuretics (18.5%), renin-angiotensin system agents (16.3%), antineoplastic agents (10.2%), and anti-inflammatory agents (5.4%). Specific drugs with high reporting odds ratios (ROR), above 10, included gentamicin, eplerenone, spironolactone, candesartan, cisplatin, acyclovir and sevoflurane [7]. In Japan, the JADER database identified frequent reports of valaciclovir hydrochloride, eldecalcitol, edaravone, and acyclovir [8]. Immune checkpoint inhibitors, such as nivolumab and ipilimumab, were also highlighted as requiring careful monitoring to prevent potential immune-related adverse events [9]. VigiBase reports suggested nephrotoxicity signals with remdesivir for COVID-19, recommending renal function assessment before and during treatment [10]. These findings indicate significant variability in drug-related AKI reporting across countries, justifying additional studies. The few studies that have used the Portuguese pharmacovigilance database (PPVD) have focused on specific drug classes [11–13].

### Aim

This study aimed to identify and estimate disproportionality rates associated with the drug classes that are most frequently reported to induce AKI, utilizing the PPVD.

### Ethics approval

The study obtained approval from the Ethics Committee for Human Research of the Faculty of Pharmacy of the University of Lisbon. (Ref: 02.2022) in 22.01.2022. According to the Portuguese legislation, the use of informed consents is waived in retrospective studies resorting to secondary data whenever data are anonymized.

## Method

### Study design

A case/non-case study was developed in 2022 using records extracted from the PPVD for the period between 01/01/2009 and 12/31/2020 [14]. Cases were identified using the ‘Acute Renal Failure’ standardized Medical Dictionary for Regulatory Activities (MedDRA) query (SMQ) [12, 14]. This SMQ was chosen so that AKI cases coded under another MedDRA term would not be omitted (Supplementary File). During the same period, all remaining reports were considered non-cases [14–16], and a random extraction without replacement of 4 non-cases per case was made [17].

### Population and sample

The Portuguese National Pharmacovigilance System (P-NPS) was created to enable adverse events suspected to be triggered by exposure to a given drug to be centrally reported and subsequently investigated for causality assessment. The P-NPS has adapted to the pharmacovigilance European Union requirements and is currently a robust system responsible for assessing the safety profile of marketed drugs and trigger actions to reduce their risks [18].

### Classification of ADRs

From each Individual Case Safety Report (ICSR), sociodemographic data, ADRs-related data (*e.g.*, descriptive term using MedDRA, date of reaction onset, and outcome), and suspected drug were extracted. ADRs are considered severe if life threatening, fatal, persistent or significant disabling, leading to or prolonging hospitalization, leading to congenital anomaly or clinically important [18]. All reports available in the PPVD occurring during the study period were considered.

### Study outcomes

The composite outcome, AKI, was defined based on the Acute Renal Failure (ARF) SMQ description, according to which ARF is characterised by a relatively rapid decline in renal function that leads to the accumulation of water, crystalloid solutes, and nitrogenous metabolites in the body [18]. Other clinical features considered for its classification include an increase in creatinine and azotaemia greater than 0.5 and 10 mg per decilitre, respectively; oliguria; and changes in the rate of urine flow. ARF may present with an onset in individuals whose baseline renal function was within normal limits. Additionally, AKI may consist of acute exacerbation of pre-existing chronic renal insufficiency [19–22]. In the PPVD, reports of AKI were identified using MedDRA dictionary considering the Preferred Terms (PT) described in Appendix 1 (Supplementary File).

### Analysed drugs

Only drugs considered 'suspected' in at least five cases of AKI were analyzed, following the methodology of Pierson-Marchandise et al. [7]. The conservative Haldane correction method was used to address cases where the involved value was zero. The Reporting Odds Ratio (ROR) was used to measure disproportionality. For drug combinations, each active substance was counted individually, except for combinations used in clinical practice such as amoxicillin and enzyme inhibitors; ceftolozane and beta-lactamase inhibitors; imipenem and enzyme inhibitors; piperacillin and enzyme inhibitors; progestogens and estrogens; and sulfamethoxazole and trimethoprim. We also analyzed the number of ‘suspected drugs’ involved in each reported AKI case and the outcome of the AKI.

### Statistical analysis

Data were coded, entered, and analysed with STATA SE v.15.1 [23]. Descriptive analysis were performed for sociodemographic characterisation, ADRs-related features (descriptive term using MedDRA, date of onset reaction, and outcome) and suspected drugs involved (according to the Anatomical Therapeutic Chemical (ATC) classification [24]). The severity criteria classified by the PPVD were used to explore a possible relationship between the ADR outcome and the case/non-case classification. Sample characterization was performed using descriptive statistics. Categorical variables were compared between groups using the chi-square (χ^2^) test. Continuous variables were compared using two independent-samples t-test with equal variances assumed. Bi-variate analysis were performed to identify associations between drugs use and AKI occurrence. The association between AKI and drug exposure was assessed as the ROR and its 95% confidence interval (CI) [25]. The significance of ROR was valued first at the ATC2 (therapeutic subgroup) level, aiming to identify drug class effects and then subsequently at the ATC5 level to evaluate if there were any specific drugs with potential safety concerns, even though no class effect had been identified. The disproportionality analysis to estimate the ROR used two-by-two contingency tables to detect signals. ROR ≥ 2, the lower limit of the 95% CI of ROR > 1 and five or more cases were defined a priori as the cut-offs for relevance [14, 26].

## Results

There were 352 AKI cases reported during this 11-year period for a total of 53,505 reports received nationally, thus representing 0.7% of all reports. Nearly all cases were reported by healthcare professionals (96.6%).

Most reports pertained to females (n = 971; 58.0%) and gender was significantly associated with the classification of AKI case (*p* < 0.001). Mean age of AKI cases (59.56 ± 21.6) was significantly higher than of non-cases (50.00 ± 22.1) (*p* < 0.001).

Among the 1760 records analysed, outcome classification was only present in 1313 reports (335 cases and 978 non-cases). In both cases and non-cases, the most common outcome classifications were clinically important, followed by hospitalization. Only hospitalization and disability were significantly associated with case/non-case attribution (*p* < 0.001). A total of 32 cases of AKI (9.6%) resulted in death. Although most reports involved only one suspected drug, it was significantly more common among AKI cases to have two or more suspected drugs involved in the report, compared to non-cases (32.4% cases vs 17.6% non-cases) (*p* < 0.001) (Table 1).

**Table 1:** Characteristics of patients, source of report and event characteristics

Characteristics	Cases	Non-cases		P-value
	AKI^a^ (n = 352) n (%)	No AKI (n = 1408) n (%)		
Age				
Years (mean ± SD)	(59.56 ± 21.6)^b^	(50.00 ± 22.1)^c^	31.776	< 0.001^1^
Sex				
Female	148 (46.1)^d^	823 (60.9)^e^	23.2272	< 0.001^2^
Male	173 (53.9)^d^	529 (39.1)^e^		
Outcome^f^				
Death	32 (9.6)	69 (7.1)	2.1911	0.139^2^
Hospitalization	170 (50.8)	261 (26.7)	65.5033	< 0.001^2^
Clinically important	215 (64.2)	639 (65.3)	0.1472	0.701^2^
Life-threatening	26 (7.8)	82 (8.4)	0.1284	0.720^2^
Disability	10 (3.0)	85 (8.7)	12.1049	0.001^2^
Congenital anomaly	1 (0.3)	0 (0.0)	2.9216	0.087^2^
‘Suspect’ drugs per event			45.771	< 0.001^2^
1 Drug (reference)	238 (67.6)	1161 (82.5)		
2 Drugs	65 (18.5)	170 (12.1)		
3 Drugs	32 (9.1)	44 (3.1)		
4 Drugs	8 (2.3)	16 (1.1)		
5 ≥ Drugs	9 (2.6)	17 (1.2)		

AKI: Acute kidney injury

^1^Independent-sample t-test; ^2^Pearson χ^2^ test

Data are n (%) unless other wise indicated

^a^AKI was the primary outcome in this study, which was defined with a narrow Standardized MedDRA Query of acute renal failure

^b^In Cases there were 150 (42.6%) reports with missing age

^c^In Non-cases there were 321 (22.7%) reports with missing age

^d^In Cases there were 31 (8.8%) reports with missing sex

^e^In Non-cases there were 56 (3.9%) reports with missing sex

^f^Each report can have more than one outcome

In total, 559 different drugs were considered 'suspect' in cases of AKI. Only three therapeutic subgroups (ATC2) showed a significant ROR: antithrombotic agents (ROR 6.72; 95% CI 2.23–20.22), antivirals for systemic use (ROR 4.02; 95% CI 2.76–5.87), and antineoplastic drugs (ROR 2.14; 95% CI 1.48–3.11). For antithrombotic and antineoplastic agents, a single drug accounted for the significance: dabigatran etexilate (ROR 3.23; 95% CI 1.35–7.72) and everolimus (ROR 44.71; 95% CI 2.57–777.14), respectively. In the antiviral class, two drugs had significant RORs: tenofovir disoproxil (ROR 3.83; 95% CI 2.08–7.06) and emtricitabine (ROR 3.14; 95% CI 1.53–6.45). Although no overall class effect was identified, three immunosuppressants showed significant RORs: mycophenolic acid (ROR 4.45; 95% CI 1.62–12.20), ciclosporin (ROR 23.65; 95% CI 6.51–85.85), and tacrolimus (ROR 3.68; 95% CI 1.02–13.25). Additionally, we identified unique drugs with significant RORs where no class effect was observed: simvastatin (ROR 8.75; 95% CI 1.71–44.72), prednisolone (ROR 11.08; 95% CI 1.24–99.15), vancomycin (ROR 9.00; 95% CI 2.80–28.96), and deferasirox (ROR 133.00; 95% CI 2.19–8082.55). In total, eleven drugs were identified, suggesting a possible association with the occurrence of AKI (Table 2).

**Table 2:** Drugs involved in AKI events and respective ROR

ATC classification	Active substance (INN)	Total number of ADRs	Total number of ADRs	ROR	[95% CI]
		With AKI^a^ (n = 559)	Without AKI^b^ (n = 1813)		
*Drugs used in diabetes (A10)*		28	35	1.18	[0.56–2.49]
	Metformin	12	20	0.56	[0.21–1.54]
	Sitagliptin	5	4	1.68	[0.41–6.98]
***Antithrombotic agents (B01)***		***34***	***62***	**6.72**	**[2.23–20.22]**
	**Dabigatran etexilate**	**20**	**19**	**3.23**	**[1.35–7.72]**
*Diuretics (C03)*		12	20	1.15	[0.52–2.54]
	Furosemide	5	4	2.86	[0.58–13.96]
*Agents acting on the renin-angiotensin system (C09)*		25	39	1.33	[0.71–2.49]
	Valsartan	7	4	3.40	[0.88–13.17]
*Lipid modifying agents (C10)*		11	24	0.83	[0.38–1.83]
	**Simvastatin**	**7**	**4**	**8.75**	**[1.71–44.72]**
*Corticosteroids for systemic use (H02)*		10	29	3.10	[0.35–27.66]
	**Prednisolone**	**9**	**13**	**11.08**	**[1.24–99.15]**
*Antibacterials for systemic use (J01)*		36	140	0.64	[0.42–0.97]
	**Vancomycin**	**9**	**5**	**9.00**	**[2.80–28.96]**
*Antimycotics for systemic use (J02)*		8	16	1.40	[0.59–3.34]
	Amphotericin B	5	12	0.56	[0.09–3.44]
***Antivirals for systemic use (J05)***		***121***	***178***	**4.02**	**[2.76–5.87]**
	**Tenofovir disoproxil**	**38**	**19**	**3.83**	**[2.08–7.06]**
	**Emtricitabine**	**24**	**13**	**3.14**	**[1.53–6.45]**
	Tenofovir alafenamide	5	1	7.63	[0.88–66.14]
	Efavirenz	7	12	0.85	[0.32–2.22]
	Sofosbuvir	6	30	0.26	[0.10–0.64]
	Sofosbuvir and ledipasvir	6	28	0.28	[0.11–0.70]
***Antineoplastic Agents (L01)***		***92***	***196***	**2.14**	**[1.48–3.11]**
	Erlotinib	6	6	2.21	[0.69–7.05]
	**Everolimus**^c^	9	0	**44.71**	**[2.57–777.14]**
	Sunitinib	5	3	3.70	[0.86–15.82]
*Immunosuppressants (L04)*		52	178	0.85	[0.58–125]
	**Mycophenolic acid**	**9**	**8**	**4.45**	**[1.62–12.20]**
	Adalimumab	9	27	1.17	[0.51–2.68]
	**Ciclosporin**	**15**	**3**	**23.65**	**[6.51–85.85]**
	**Tacrolimus**	**5**	**5**	**3.68**	**[1.02–13.25]**
***All other therapeutic products (V03)***		9	3	**46.50**	**[6.7–322.62]**
	**Deferasirox**^c^	9	0	**133.00**	**[2.19–8082.55]**

ADR, Adverse Drug Reaction; ATC, Anatomical, Therapeutic and Chemical; AKI, Acute Kidney Injury; CI, Confidence Interval; PPVD, Portuguese National Pharmacovigilance Database; INN, International Nonproprietary Name; ROR, Reporting Odds Ratio; Bold, indicates the subgroups (ATC2) and drugs (ATC5) evidencing a significant ROR

^a^In cases there were 23 (4.1%) reports with missing ATC5

^b^In non-cases there were 127 (7.0%) reports with missing ATC5

^c^Haldane correction statistical method

## Discussion

### Statement of key findings

The main findings suggest the drug classes most frequently associated with AKI were antithrombotic agents (B01), namely dabigatran, antivirals for systemic use (J05), including tenofovir disoproxil and emtricitabine, and antineoplastic agents (L01), like everolimus. Additionally, other medications linked to AKI included simvastatin, prednisolone, vancomycin, mycophenolic acid, cyclosporin, tacrolimus and deferasirox.

Previous studies have linked AKI to systemic antibacterials, antineoplastics, analgesics (N02), and immunosuppressants [27–36]. Our findings were consistent, except for analgesics. We observed disproportionality for antithrombotic agents (B01), particularly dabigatran, which has not been previously reported.

In our study, simvastatin was the only statin identified. Although generally well-tolerated, the most common complication of statins is myopathy, affecting up to 7% of patients on long-term treatment [37].

Prednisone was associated with AKI, possibly related to 'renal scleroderma crisis of unknown frequency', although its use in conditions prone to AKI, making causal interpretation challenging.

Antivirals such as tenofovir disoproxil, a nephrotoxic nucleoside reverse transcriptase inhibitor, which can cause AKI, with or without proximal tubulopathy [38] and emtricitabine, were both associated with an elevation in creatine kinase. Desferasirox, with a known dose-dependent nephrotoxic effect, was also identified with a high ROR, but which may be influenced by the patient’s iron status.

Several well-documented nephrotoxic drugs were identified, including vancomycin, everolimus and calcineurin inhibitors (cyclosporine and tacrolimus). These effects are biologically plausible [39], and are consistent with previous studies using the FPVD, except for tenofovir disoproxil, for which its nephrotoxicity is well-established [7]. The immunosuppressant mycophenolic acid is not typically directly linked to AKI but is often used alongside calcineurin inhibitors, which carry an increased risk of AKI.

### Strengths and weaknesses

An important strength of this study is the use of a national database, the robust methods used, including the use of statistical tools recognized for their value in pharmacovigilance. Such studies support the value of clinical pharmacy monitoring [40]. Nonetheless, there are also limitations worth noting. One is associated with the case/non-case methodology using spontaneous notifications, which does not allow for accurate estimation of the true incidence of ADRs due to reporting bias, specifically underreporting [41]. However, this underreporting does not significantly impact the results of this study. Another limitation concerns data quality, as spontaneous reporting data were not subject to additional clinical assessment or qualitative validation. The longer market presence of some drugs compared to others may also introduce selection bias. Additionally, the limitations related to the exhaustiveness of available variables include the inability to evaluate treatment indications, prescribed doses, or treatment duration, all of which may influence the outcome and contribute to misclassification bias. This is relevant for drugs requiring dose adjustments in renal insufficiency, such as dabigatran or deferasirox [42]. The absence of clinical information further exacerbates this limitation, as it could have helped to control confounding factors such as chronic conditions (e.g. CKD, cardiovascular disease, chronic liver disease), and metabolic disorders (e.g. anemia, hypoalbuminemia, obesity) [1, 5, 43–45]. The Weber effect may have influenced reports on prednisone, given that ADRs described as “of unknown frequency” in the SmPC are more likely to be reported. Finally, despite using a national database, Portugal's relatively small size and the rarity of AKI can lead to focusing on very small numbers, affecting confidence intervals and certainty.

### Interpretation

When investigating the drugs that demonstrated disproportionality, we identified that for dabigatran etexilate, the SmPC does not list renal adverse events as known effects, but renal insufficiency can be a contraindication or factor for considering non-use or dose reduction of Non-Vitamin K Oral Anticoagulants (NOACs) due to bleeding risks. While the frequency is unknown, renal complications can arise from severe bleeding, such as compartment syndrome and renal failure due to hypoperfusion or anticoagulant-associated nephropathy. In 2018, Caldeira et al. conducted a retrospective study analyzing safety reports related to oral anticoagulants in the PPVD over five years, identifying moderate disproportionality in the 'Nervous system disorders' system organ class (SOC), specifically linked to NOACs [12]. It is important to conduct further studies on dabigatran etexilate to analyze whether the effect is dose-dependent, whether it occurs only in cases of bleeding, and whether it is a characteristic of the class.

In the case of statins, the myopathy observed as a complication can range from nonspecific myalgia without elevated creatine kinase to rhabdomyolysis, a rare but serious condition that involves muscle necrosis and the release of muscle components into the bloodstream. Severe rhabdomyolysis can lead to ARF and even death [46]. Therefore, the effect identified is pharmacologically plausible. Although not renally eliminated, it remains unclear if simvastatin’s effect depends on renal function, or if it only occurs in patients who manifest rhabdomyolysis.

The nephrotoxic mechanism of tenofovir disoproxil is well-documented. AKI may result from direct tubular toxicity mediated by mitochondrial injury, leading to acute tubular necrosis [47]. Proximal tubular dysfunction may cause electrolyte imbalances, including Fanconi syndrome. While withdrawal can reverse the condition, some patients may progress to chronic kidney disease [48, 49].

There is absent literature about emtricitabine’s association withn AKI, even though the SmPC mentions that the elevation of creatine kinase is very frequent. Among antivirals for systemic use, tenofovir and emtricitabine showed ROR of the same order of magnitude, with CIs having little dispersion. This suggests some consistency, and since emtricitabine is less well-known for its risk of inducing AKI, further research is needed to confirm whether this risk is dose-dependent.The increased risk of AKI with high-dose deferasirox and lower serum ferritin suggests that overchelation may contribute to toxicity. This finding highlights the need for close monitoring of renal function and serum ferritin, using the lowest effective dose, and considering discontinuation of deferasirox if AKI or volume depletion is suspected [50]. The SmPC for deferasirox mentions it has not been studied in patients with renal impairment and is contraindicated in those with a creatinine clearance below 60 ml/min. Post-marketing reports include cases of ARF requiring dialysis. Regular monitoring of serum creatinine, serum ferritin, and transaminase levels is recommended to track trends. A study by Bird et al. describes this dose-dependent mechanism for AKI, reinforcing the need for dose monitoring in clinical practice [50]. Notwithstanding, it should be stressed that deferasirox exhibits a very large dispersion in its ROR, conferring lower certainty. Although mycophenolic acid is generally not seen as a primary cause of AKI, careful monitoring and further prospective studies are needed, especially in patients on multiple immunosuppressants or with pre-existing kidney conditions, to validate the disproportionality observed in our study, mostly given the absence of safety and efficacy data in patients having undergone liver or heart transplants.

Despite the lack of statistical significance, it is important to note that nearly 10% of AKI cases resulted in death in our study. Understanding real-world data is crucial for assessing the risks associated with medications, as some studies indicate that, depending on the definition used and on the study design, drugs are associated with AKI in 19–37.5% of adults [3, 5, 51, 52].

Previous studies have suggested that involving clinical pharmacists in medication review and medicines optimization may be crucial to increase patient safety [53, 54]. In this context, our findings highlight the crucial role of clinical pharmacy activities in close monitoring of renal function of patients with known risk factors or those prescribed medications that increase the risk of AKI.

### Further research

In case/non-case studies, the ROR corresponds to the risk of spontaneous reporting of an ADR and not the risk of the occurrence of the ADR itself. This association may be reduced if the medication has been consistently linked to a specific reaction, leading to an increase in the frequency of non-cases. It is important to note that these associations suggest a potential link between these medications and the occurrence of AKI, but they do not confirm a causal relationship. Further studies would be necessary to establish causality. Moreover, clinical validation may be necessary to establish causation and understand the underlying mechanisms. Additionally, the methodology used can only generate hypothesis, which should be confirmed through subsequent experimental studies. Considering the database used is national, it would be relevant to also identify the drug classes most frequently reported as inducing AKI at an European or even at a global level, utilizing databases such as EudraVigilance or Vigibase. This would enable verifying variations in the incidence of reported ADRs between the different countries.

## Conclusion

In this study, we highlight the identification of dabigatran etexilate**,** simvastatin**,** emtricitabine**,** and mycophenolic acid as being implicated in AKI through disproportionality analysis of ADR reports over an 11-year period in PPVD. While vancomycin, tenofovir disoproxil, everolimus, ciclosporin, and tacrolimus are mostly well-known for their association with AKI, this study highlights the importance of clinical pharmacy activities in closely monitoring the renal function of patients prescribed medications known to increase the risk of AKI. For the drugs not previously reported we stress the need to monitor renal function in patients with known risk factors, whilst investing in further research to confirm hypotheses raised.

## Supplementary Information

Below is the link to the electronic supplementary material.

Supplementary file1 (DOCX 68 kb)
