# Supplementary figures and images for: A case/non-case study of a national pharmacovigilance database to explore drug-induced acute kidney injury
Source: Int J Clin Pharm. 2025 May 26;47(6):1730–8. doi: 10.1007/s11096-025-01940-0 (PMC12630246; doi:10.1007/s11096-025-01940-0)

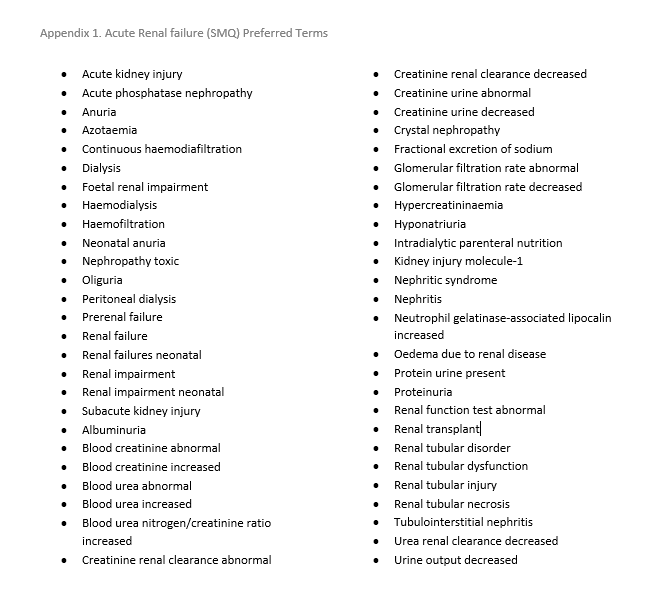

Supplement: Supplementary file 1 — Supplementary file1 (DOCX 68 kb) [file 11096_2025_1940_MOESM1_ESM.docx]
